# Update on the Kelch-like (KLHL) gene family

**DOI:** 10.1186/1479-7364-7-13

**Published:** 2013-05-15

**Authors:** Bajinder S Dhanoa, Tiziana Cogliati, Akhila G Satish, Elspeth A Bruford, James S Friedman

**Affiliations:** 1 Department of Ophthalmology, University of Colorado School of Medicine, Aurora, CO 80045, USA; 2 Neurobiology-Neurodegeneration and Repair Laboratory (N-NRL), National Eye Institute, National Institutes of Health, Building 6/302, MSC 0610, 6 Center Drive, Bethesda, MD 20892, USA; 3 HUGO Gene Nomenclature Committee (HGNC), EMBL-EBI, Wellcome Trust Genome Campus, Hinxton, Cambridgeshire CB10 1SD, UK

**Keywords:** KLHL, Kelch domain, BTB domain, Ubiquitination, Gene family, Evolution, Mendelian disease, Gene nomenclature, Cancer

## Abstract

The Kelch-like (KLHL) gene family encodes a group of proteins that generally possess a BTB/POZ domain, a BACK domain, and five to six Kelch motifs. BTB domains facilitate protein binding and dimerization. The BACK domain has no known function yet is of functional importance since mutations in this domain are associated with disease. Kelch domains form a tertiary structure of β-propellers that have a role in extracellular functions, morphology, and binding to other proteins. Presently, 42 KLHL genes have been classified by the HUGO Gene Nomenclature Committee (HGNC), and they are found across multiple human chromosomes. The KLHL family is conserved throughout evolution. Phylogenetic analysis of KLHL family members suggests that it can be subdivided into three subgroups with KLHL11 as the oldest member and KLHL9 as the youngest. Several KLHL proteins bind to the E3 ligase cullin 3 and are known to be involved in ubiquitination. KLHL genes are responsible for several Mendelian diseases and have been associated with cancer. Further investigation of this family of proteins will likely provide valuable insights into basic biology and human disease.

## Introduction

The KLHL (Kelch-like) gene family encodes proteins that constitute a subgroup at the intersection between the BTB/POZ domain and Kelch domain superfamilies. First identified in the vaccinia virus [1], the BTB motif was named based on a homologous domain of 115 amino acids in *Drosophila melanogaster* bric à brac 1, tramtrack, and broad-complex proteins (BTB) [2]. BTB domain-containing zinc finger proteins are conserved evolutionarily from *Drosophila* to humans and mice with more than 49 family members in the latter two [3]. The POZ domain was initially named after the 120 amino acid motifs present at the amino terminus in poxvirus proteins and zinc finger proteins [4]. The BTB/POZ domain facilitates protein binding [5] as reviewed in Perez-Torrado et al. [6]. Functions observed to be associated with other BTB-containing proteins involve a variety of cellular mechanisms such as control of cytoskeletal organization [7], ion channel gating [8], transcription suppression [9], and protein targeting for ubiquitination through cullin E3 ligases [10,11].

The Kelch repeat or domain is also an evolutionarily conserved structure that can be found from *Drosophila melanogaster* to *Homo sapiens*[12]. Based on sequence identity, it has been suggested that each four-stranded β-sheet of the Kelch motif forms one blade of a β-propeller structure [12]. The Kelch superfamily of proteins can be subdivided into five groups. These subgroups include (1) N-propeller, C-dimer proteins, (2) N-propeller proteins, (3) propeller proteins, (4) N-dimer, C-propeller proteins, and (5) C-propeller proteins [12]. Kelch-containing proteins have roles in extracellular communication/interaction, cell morphology, gene expression, actin binding, and can be co-opted by virus post-infection [12]. KLHL family members belong to the N-dimer, C-propeller subclass of Kelch repeat proteins.

In addition to BTB/POZ and Kelch domains, the KLHL family members contain a BACK domain, first described as a 130-residue region of conservation observed amongst BTB-Kelch proteins [13]. Although no function has been assigned to the BACK domain, it is likely to be of functional significance because mutations in this region have been shown to cause human disease [14-19].

The BTB superfamily includes KLHL, KBTBD, and KLHDC subfamilies, which encompass structurally related molecules that differ in the types and numbers of their protein domains. In general, KLHL proteins contain one BTB/POZ domain, one BACK domain, and five to six Kelch motifs (BTB, BACK, 5/6 Kelch). KBTBD proteins have one BTB/POZ domain, occasionally a BACK domain, and two to four Kelch motifs (BTB/POZ, (BACK), 2/4 Kelch). Most KLHDC family members contain only three to seven Kelch motifs and usually do not have BTB or BACK domains (3/7 Kelch). Herein, we describe the evolutionary structure of the human KLHL family, discuss examples of KLHL family member biology, and present an overview of KLHL protein relationship to human disease.

### Evolutionary structure of the human KLHL family

There are nine KBTBD genes in the human genome, and their encoded proteins typically possess a BTB and BACK domain and two to four Kelch motifs. Ten KLHDC genes have been defined by the HUGO Gene Nomenclature Committee (HGNC) in the human genome. KLHDC proteins generally do not have BTB or BACK domains and have three to seven Kelch motifs. These subfamilies will not be discussed further in this review.

**Figure 1:**
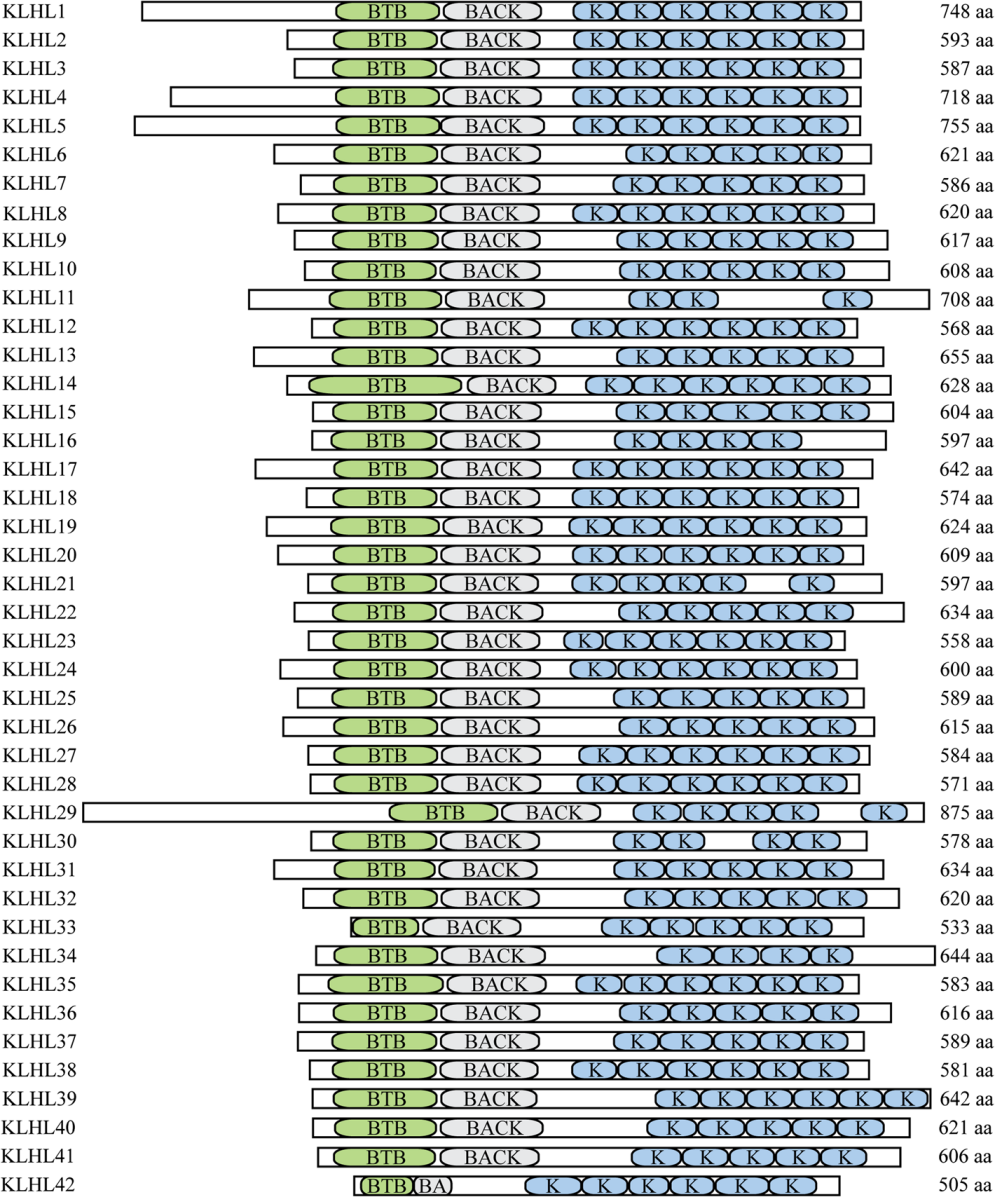
**Schematic diagram of KLHL family members drawn to scale with amino acid numbers listed on the right.** ‘BTB,’ ‘BACK,’ and ‘K’ indicate the BTB domain, BACK domain, and Kelch domains, respectively. In case of multiple isoforms, only the first one is depicted. Data was collected from [20] using a motif scan with Pfam HMMs (global or local models).

KLHL proteins generally have a BTB/POZ domain, a BACK domain, and five to six Kelch domains. However, the domain composition for this family can appear to vary depending on the protein domain prediction program used to examine their protein sequences. The KLHL family protein structure is shown in Figure 1. It should be noted that KLHL29, KLHL31, KLHL40, and KLHL41 were previously assigned within the KBTBD family and were listed as KBTBD1 (KLHL31), KBTBD5 (KLHL40), KBTBD9 (KLHL29), and KBTBD10 (KLHL41). When these genes were originally named, they appeared to encode less than the five to six Kelch domains in the standard KLHL (BTB, BACK, 5/6 Kelch) structure. Therefore, they were classified as part of the KBTBD family; however, reannotation has shown that these genes do encode 5/6 Kelch domains. Likewise, the reannotation of KLHDC5 (KLHL42) has shown that it encodes the standard KLHL (BTB, BACK, 5/6 Kelch) structure. Consequently, they have since been placed within the KLHL family.

**Figure 2:**
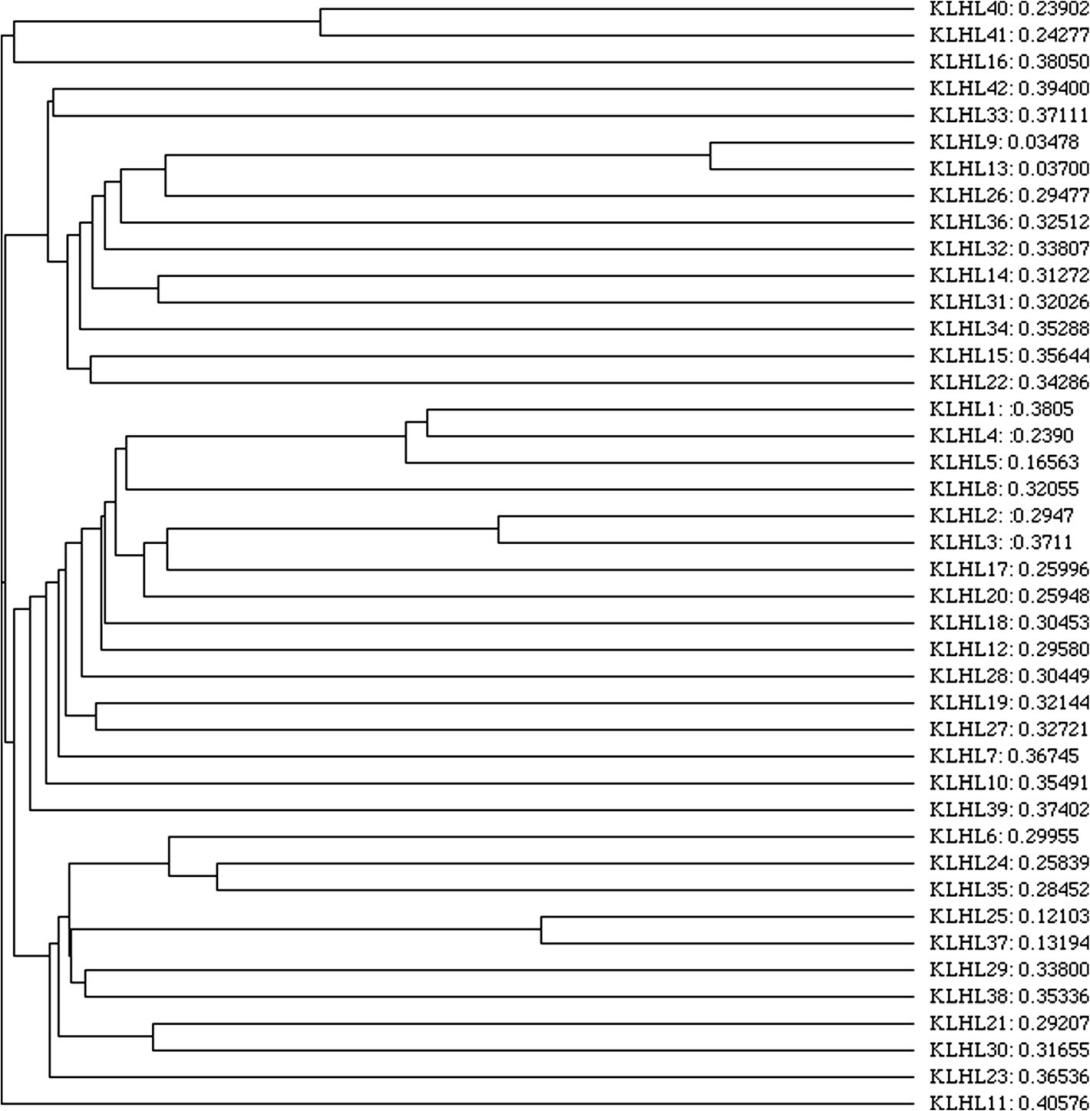
**Phylogenetic tree of KLHL proteins with evolutionary distances shown next to each protein.** Amino acid sequences were obtained from [21], and the alignment was created with ClustalO from [22]. The Cladogram was created using ClustalW2-Phylogeny from [23].

The HGNC presently defines 42 KLHL genes. They are spread over different chromosomes, though several are located on chromosomes 1, 4, and X (Additional file 1). The number of exons is not conserved and ranges from a single coding exon to 15 coding exons. However the number of KLHL genes is conserved between mammalian species (e.g., *Homo sapiens* and *Mus musculus*). ClustalO and phylogenetic analyses of KLHL family members suggest that they can be subdivided into multiple subgroups (Figure 2). KLHL11 appears the oldest to diverge, followed by KLHL42 and KLHL16. The most recent divergences appear to have happened for KLHL9 and KLHL13, followed by KLHL25 and KLHL37 (Figure 2).

### Examples of KLHL family members

#### KEAP1 (KLHL19)

The *KEAP1* gene is clearly a member of the KLHL family. While the well-used KEAP1 symbol has been retained as the official gene nomenclature; it has also been assigned the KLHL19 synonym to clearly group it within the KLHL family. The human *KEAP1* (Kelch-like ECH-associated protein 1 gene is located on chromosome 19p13, and the mouse ortholog is located on chromosome 9. KEAP1 was originally identified as a nuclear factor erythroid 2-related factor 2 (NRF2) interacting protein. NRF2 is a transcription factor that is essential for the cellular reaction to electrophiles and binds to the antioxidant response element (ARE) present in promoters of genes involved in phase II detoxifying and oxidative stress enzyme response [24]. KEAP1 was shown to negatively control NRF2 transactivation potential [25]. Some of the genes controlled by NRF2 are important for cellular defense against harmful oxidative stresses that could result in carcinogenesis (as reviewed in Motohashi and Yamamoto [26]). NRF2 interacts as a heterodimer with Maf transcription factor protein that binds to the ARE and activates transcription through the Maf recognition element (MARE) (reviewed in Motohashi and Yamamoto [26]).

KEAP1's ability to repress NRF2's downstream target activation was demonstrated in zebrafish by gain-of-function and loss-of-function experiments in which transcriptional control by NRF2 of glutathione S-transferase gene (*gstp)* induction was tested in the absence and presence of KEAP1. NRF2-induced *gstp* expression was repressed when KEAP1 was also overexpressed [27]. KEAP1 was found to promote NRF2 degradation by targeting it for ubiquitination through the cullin 3 ligase complex [28,29], thus preventing NRF2 activity under normal cellular conditions [30]. Domain function analysis of KEAP1 revealed that NRF2 degradation requires both BTB and intervening domains [28].

KEAP1 is localized primarily in the cytoplasm of cells, with a minimal amount in the nucleus and endoplasmic reticulum [31]. KEAP1 scaffolding to the actin skeleton is crucial for effective sequestering of NRF2 into the cytoplasm [7]. Electrophiles promote the nuclear accumulation of NRF2 without altering the subcellular localization of KEAP1 [31]. Furthermore, KEAP1 appears to regulate both translational and post-translational responses to oxidative stress [32,33].

The crystal structure of mouse KEAP1 was resolved to reveal a six-bladed propeller tertiary structure encompassing the Kelch domain [34]. The analysis showed which bonds are needed for structural integrity and proper association between KEAP1 and NRF2 [34]. NRF2 has been suggested to be involved in susceptibility of lungs to cigarette smoke through the induction of antioxidant genes [35]. KEAP1's relationship with cancer is described below.

#### KLHL2 (Mayven)

KLHL2/Mayven is an actin-binding protein that shares 63% identity (77% similarity) with the *Drosophila* ring canal (Kelch) protein [36]. The human gene is localized on chromosome 4q21.1, and its murine ortholog is on chromosome 8 [36]. *KLHL2* RNA can be detected in developing and adult brain, heart, spleen, lung and liver, and adult kidney but most predominantly throughout the brain including the hippocampus, caudate nucleus, corpus callosum, and amygdala [36]. The KLHL2 protein has been shown to be constitutively expressed in developing and mature oligodendrocytes and neurons where it binds directly to F-actin through its Kelch repeats and plays an important role in the organization of the actin cytoskeleton [36,37]. In developing oligodendrocytes, KLHL2 is upregulated during differentiation [38], and it localizes to lamellipodia (but not filipodia) [39]. KLHL2 overexpression in oligodendrocyte progenitors accelerates neurite outgrowth and leads to longer processes. Conversely, downregulation inhibits process extension [37,38]. KLHL2 co-localizes and associates with FYN oncogene related to SRC, FGR, and YES (FYN) tyrosine kinase through its SH3-binding domain at the N-terminus. This interaction increases upon differentiation, suggesting a role in promoting oligodendrocyte process outgrowth and strengthening of the initial axonal-glial contact mediated by FYN signaling. Based on the type of interaction, it has been suggested that KLHL2 acts as a linker between FYN and actin [38]. Upon depolarization of primary hippocampal neurons with KCl, association of KLHL with actin is enhanced, resulting in re-distribution and translocation along the axonal processes [36]. The functional significance of these findings needs further investigation.

Recently, independent interactions with two different proteins have underscored KLHL2 involvement in ubiquitination. KLHL2 binds to nucleus accumbens-associated 1 (NACC1), a transcriptional repressor protein that interacts also with another KLHL family member, ectodermal-neural cortex 1 (ENC1/KLHL37). KLHL2 may form part of the ubiquitin complex through which NACC1 affects degradation of specific gene products and promotes proteasome activity and trafficking [40]. Finally, interactions of KLHL2 have been shown with neuronal pentraxin with chromo domain (NPCD, encoded by the *Npcd* gene in mouse which is a read-through between *Cbx6* and *Nptxr*) and with cullin 3. In this case, KLHL2 appears to function as a specific adapter for NPCD ubquitination via cullin 3. Notably, overexpression of NPCD in hippocampal neurons leads to apoptosis [41].

### KLHL family members and inherited disease

KLHL members associated with inherited forms of human disease include KLHL3, KLHL7, KLHL9, KLHL12, and GAN (KLHL16). Mutations in *KLHL3* have been identified in patients with familial hyperkalemic hypertension. Molecular analyses have shown that most mutations increase the activity of the Na^+^Cl^−^ symporter in the distal convoluted tubule portion of the nephron. The increased activity causes more Na^+^ and Cl^-^ reabsorption, increasing blood pressure and culminating in hypertension. Although mutations were identified in this familial disease, screening for single nucleotide polymorphisms (SNPs) in *KLHL3* did not reveal any significant association with blood pressure measurements in human subjects, indicating that common variants are not responsible for variations in blood pressure [19].

Patients with autosomal dominant retinitis pigmentosa (adRP), a neurodegenerative disease that leads to loss of rod and cone photoreceptors, carry mutations in *KLHL7*[18]. Early and often rapid alteration of rod function is a characteristic of the classic form of adRP, but the phenotype observed in the *KLHL7* patients differs because of its late onset and preserved rod function in older family members [42,43]. Recent work suggests that KLHL7 protein binds to cullin 3 and that a single mutation in the BACK domain leads to reduced efficiency of cullin 3's ubiquitin ligase activity [44]. KLHL7 and KLHL12 have also been identified as autoantigens in Sjögren's syndrome, an autoimmune disease that causes damage to the salivary and lachrymal glands [45]. No significant immunological response to either KLHL7 or KLHL12 was observed in the sera of healthy individuals [45].

A missense mutation in *KLHL9* leads to the development of distal myopathy in human patients. Distal myopathy is a degenerative disease that initially causes atrophy of distal limb muscles and subsequently extends to proximal limbs [46]. Mutations in GAN (KLHL16), or gigaxonin, are linked to human giant axonal neuropathy, an autosomal recessive disorder [14]. Giant axonal neuropathy affects the central and peripheral nervous system that becomes populated with large, dysfunctional axons [16]. Compound heterozygous missense mutations in the Kelch-coding region of gigaxonin have recently been reported in Chinese cases [47].

### KLHL family members and cancer

Four KLHL family members are associated with cancer: KLHL6, KEAP1 (KLHL19), KLHL20, and ENC1 (KLHL37). Mutations in *KLHL6* are recurrent in cases of chronic lymphocytic leukemia (CLL), a type of leukemia that is predominant in adults. Six mutations were identified within *KLHL6* in CLL patients, and all disrupt KLHL6 function in germinal center B cell formation [48,49]. Insertion, missense, frameshift, and missense mutations in *KEAP1*[17,35] have been identified in cancerous cells of the liver, gallbladder, and lung. Mutations that result in KEAP1 loss of function are thought to facilitate cancer cell expansion. Decreased KEAP1 expression releases the block on the transcriptional activity of NRF2, resulting in increased expression of oxidative stress enzymes and proteins that favor cancer cells survival and proliferation [33,50]. KLHL20 is an important KLHL family member related to cancer progression. KLHL20 is induced by HIF-1α protein and forms a KLHL20-cullin 3 complex that degrades the promyelocytic leukemia protein (PML) leading to prostate cancer progression [51]. Mutations in ENC1 (KLHL37), also called NRP/B, are associated with brain tumors. Mutations reported in KLHL37 are primarily located in the Kelch domain but also exist in the BTB and BACK domains [15]. KLHL37 protein is normally expressed in neurons but not in astrocytes; however, brain tumor cells that arise from astrocytes express KLHL37. It is hypothesized that mutated KLHL37 promotes cell growth, prevents apoptosis, and alters the cytoskeleton [15].

## Conclusions

There are 42 KLHL family members encoded in the human genome, containing conserved BTB, BACK, and Kelch domains. KLHL proteins are known to be involved in the ubiquitination process, but the specific roles for each family member have not yet been elucidated. KLHL proteins will likely have multiple substrates. KEAP1 (KLHL19), for example, has at least three (NRF2, IKKβ and BCL-2) as reviewed in Tian et al. [52]. Similar protein motifs amongst substrates such as the ETGE region in NRF2 and IKKβ could be one way to explain how a KLHL protein can bind to more than one protein [52]. Another potential source of substrate diversity is possible through the BTB domain. BTB cross-dimerization between different KLHL proteins could theoretically allow for differential substrate binding depending on the spatial and temporal expression of KLHL proteins. Mutations in certain KLHL genes are detrimental and result in either Mendelian disease or human cancers. We anticipate that further studies will reveal that most, if not all, KLHL proteins have fundamental impacts on human biological processes and disease.

## Competing interests

The authors declare that they have no competing interests.

## Authors' contributions

BSD, TC, AGS, EAB, and JSF. drafted the manuscript. BSD, EAB, and JSF carried out sequence alignments and/or domain structure analyses. All authors read and approved the final manuscript.

## Supplementary Material

Additional file 1 Human KLHL member summary as recorded in the National Center for Biotechnology Information (NCBI, http://www.ncbi.nlm.nih.gov), HUGO Gene Nomenclature Committee (HGNC, http://www.genenames.org), and Online Mendelian Inheritance in Man (OMIM, http://www.omim.org) databases. Click here for file
